# Accumulation of senescence observed in spinocerebellar ataxia type 7 mouse model

**DOI:** 10.1371/journal.pone.0275580

**Published:** 2022-10-17

**Authors:** William Miller, Charles Lewis Humphrey Pruett, William Stone, Cindy Eide, Megan Riddle, Courtney Popp, Matthew Yousefzadeh, Christopher Lees, Davis Seelig, Elizabeth Thompson, Harry Orr, Laura Niedernhofer, Jakub Tolar

**Affiliations:** 1 Department of Pediatrics, Medical School, University of Minnesota, Minneapolis, MN, United States of America; 2 Institute on the Biology of Aging and Metabolism, University of Minnesota, Minneapolis, MN, United States of America; 3 Department of Biochemistry, Molecular Biology and Biophysics, College of Biological Sciences, University of Minnesota, Minneapolis, MN, United States of America; 4 Comparative Pathology Shared Resource, College of Veterinary Medicine, University of Minnesota, Minneapolis, MN, United States of America; 5 Institute for Translational Neuroscience, University of Minnesota, Minneapolis, MN, United States of America; 6 Department of Laboratory Medicine and Pathology, Medical School, University of Minnesota, Minneapolis, MN, United States of America; Universitair Medisch Centrum Groningen, NETHERLANDS

## Abstract

Spinocerebellar ataxia type 7 (SCA7) is a neurodegenerative disease caused by a trinucleotide CAG repeat. SCA7 predominantly causes a loss of photoreceptors in the retina and Purkinje cells of the cerebellum. Severe infantile-onset SCA7 also causes renal and cardiac irregularities. Previous reports have shown that SCA7 results in increased susceptibility to DNA damage. Since DNA damage can lead to accumulation of senescent cells, we hypothesized that SCA7 causes an accumulation of senescent cells over the course of disease. A 140-CAG repeat SCA7 mouse model was evaluated for signs of disease-specific involvement in the kidney, heart, and cerebellum, tissues that are commonly affected in the infantile form. We found evidence of significant renal abnormality that coincided with an accumulation of senescent cells in the kidneys of SCA7^*140Q/5Q*^ mice, based on histology findings in addition to RT-qPCR for the cell cycle inhibitors *p16*^*Ink4a*^ and *p21*^*Cip1*^ and senescence-associated ß-galactosidase (SA-ßgal) staining, respectively. The Purkinje layer in the cerebellum of SCA7^*140Q/5Q*^ mice also displayed SA-ßgal^+^ cells. These novel findings offer evidence that senescent cells accumulate in affected tissues and may possibly contribute to SCA7’s specific phenotype.

## Introduction

Spinocerebellar ataxia type 7 (SCA7) is a neurodegenerative disease caused by a genomic expansion of trinucleotide CAG repeats encoding polyglutamine (polyQ) in ATXN7. An increased number of CAG repeats causes both an increased severity of SCA7 and an earlier age of symptom onset [1, 2]. Adult-onset SCA7 is most common and results in the selective loss of the Purkinje cells of the cerebellum and the photoreceptors of the retina, leading to progressive ataxia and loss of vision [3]. Infantile-onset SCA7 causes a more varied phenotype, with hypotonia, failure to thrive, patent ductus arteriosus, hypertrophic cardiomyopathy, and nephrotic syndrome previously reported [1]. While the causative mutation and resultant phenotype of SCA7 are both known, it is unknown how the CAG expansion in ATXN7 damages Purkinje cells and photoreceptors in adult-onset SCA7s, and damages the kidney and heart as seen in infantile-onset SCA7.

Existing evidence has shown that SCA7 results in an increased susceptibility to DNA damage. Increased markers of DNA damage have been reported in multiple SCA7 cell lines, cerebellar and cortical neurons from SCA7 mouse models, and from progenitor neurons derived from SCA7 patient stem cells [4–6]. Additionally, Swintonski et al. demonstrated that polyglutamine-expanded ataxin-7 inhibits the DNA repair pathways homology-directed repair (HDR) and single-strand annealing (SSA) in a length dependent manner. These functional defects in DNA repair pathways resulted in significantly increased yH2Ax in protein-lysates from the brains of 266 CAG SCA7 mice at 8 weeks of age [6]. This increased marker of DNA damage occurred after previously reported symptom onset at 5 weeks of age, but before formation of detectable ataxin-7 microaggregates at 12 weeks of age [6]. While Swintonski et al. did not identify the specific mechanism of how polyglutamine expanded ataxin-7 impairs DNA damage, they propose that it could occur through a loss of endogenous ATXN7 function.

ATXN7 is a component of the Spt-Ada-Gcn5-Acetyltransferase (SAGA) complex. SAGA has two enzymatic modules: a histone acetyltransferase (HAT) module and a deubiquitylation (DUB) module [7]. While SAGA has traditionally been known to be a transcriptional coactivator, recent evidence has shown that SAGA plays a role in DNA repair. Clouaire et al. found that SAGA deubiquitinated H2B lysine 120 following DNA double-strand breaks [8]. Furthermore, it was reported that knockdown of Usp22 and Eny2, two components of the SAGA DUB module, impaired the DNA repair pathways non-homologous end joining (NHEJ) and HDR [9]. SAGA function is significantly impaired in SCA7 [10]. Therefore it is possible that impaired function of this complex, a known contributor to DNA repair, would cause increased DNA damage. However, it is also possible that this well-observed increased susceptibility to DNA damage in SCA7 is a downstream effect of the known protein aggregation, as aggregation has previously been correlated to increased genomic instability [11]. Whatever the cause, increased DNA damage has been consistently demonstrated in SCA7 and the effects of increased DNA damage in SCA7 are unknown.

A known consequence of increased DNA damage thought to contribute to other age-related diseases is the accumulation of senescent cells [12–14]. Cellular senescence is an irreversible arresting of the cell cycle triggered by multiple stressors, including genomic instability arising from radiation exposure, oxidative DNA damage, or through telomeric erosion [15]. The p53/p21^CIP1^ and p16^INK4a^/Rb tumor suppression pathways govern the exit of senescent cells from the cell cycle [15]. Senescent cells are morphologically distinct, swelling in size, demonstrating ß-galactosidase activity at pH 6 (SA-ßgal) [16]. Detection of ß-galactosidase activity at pH 6.0 has long been noted to be present in senescent cells and is due to the increased lysosomal content and lysosomal ß-galactosidase [17]. Additionally, senescent cells actively secrete a combination of proinflammatory chemokines, cytokines, extracellular vesicles, and other soluble factors in what is known as the senescence-associated secretory phenotype (SASP) [18]. The selective removal of senescent cells has been shown to lead to functional improvement in murine models of Alzheimer’s and Parkinson’s [19, 20].

In this paper, we hypothesized that SCA7 causes an accumulation of senescent cells in tissues most commonly affected by its phenotype, namely the cerebellum, kidney, and heart. To test this, we utilized a previously established 140 CAG knock-in murine model of infantile SCA7, derived from the commonly used 266 CAG model [21, 22]. The 140 CAG mice (SCA7^140Q/5Q^) used in this study were previously characterized as displaying weight loss, photoreceptor dysfunction, gene deregulation of Purkinje cells, Purkinje cell morphology and functional dysfunction, and defects in SAGA-dependent epigenetic marks [21]. The 266 CAG model mice have been further characterized as having an infantile SCA7 phenotype of weight loss, retinal dysfunction, Purkinje cell dysfunction, and progressive ataxia [5, 21, 23]. These phenotypes are reported in infantile SCA7 patients, in addition to nephrotic syndrome, patent ductus arteriosus, and hypertrophic cardiomyopathy [1]. However, organ defects in the kidney and heart have not previously been investigated in SCA7^140Q/5Q^ or in SCA7^266Q/5Q^ mice. Therefore, prior to investigating senescence, we examined the cerebellum, heart, and kidney for signs of disease-specific involvement. We found signs of kidney abnormalities that indicate this model may effectively exhibit SCA7 renal dysfunction, but did not identify either patent ductus arteriosus or hypertrophic cardiomyopathy. We then investigated affected tissues for changes in senescent cell burden as measured through expression of classical senescence markers (*p16*^*Ink4a*^ and *p21*^*Cip1*^) and staining for SA-ßgal and found evidence that senescent cells accumulated in the kidney and in the Purkinje layer of the cerebellum.

## Methods

### Murine model characterization

Animal research was approved by the University of Minnesota Institutional Animal Care and Use Committee under protocol #1808-36332A (which has since been renewed to be # 2108-39344A). Mice were housed using standard, small-animal research conditions per University of Minnesota IACUC guidelines with a 12-hour light:dark cycle, free access to food and water, and temperatures ranging from 22–24° C. Animals were sacrificed using the UMN IACUC approved procedure of cervical dislocation/separation.

The SCA7^*140Q/5Q*^ mouse model we utilized had an ATXN7 CAG expansion of approximately 140–150 repeats and was kept on a C57BL/6J background as previously described [21]. Blinded phenotypic ataxia analysis was performed using a previously published protocol [24] (S1 Fig). Briefly, mice are scored on four measurements of coordination: coordination on ledge of a cage, hindlimb clasping, tremor when walking, and kyphosis. Each measurement was scored on a scale of 0–3, with a 0 indicating no relevant phenotype and a 3 indicating a severe phenotype. Scores were added. SCA7^*140Q/5Q*^ and wild-type (WT) mice were weighed longitudinally (S1 Fig). Mice were housed using standard, small-animal research conditions per University of Minnesota IACUC guidelines with a 12-hour light:dark cycle, free access to food and water, and temperatures ranging from 22–24° C. Genotyping was performed via PCR using a previously established protocol [22]. Endpoints for experimentation were chosen in accordance with previously published survival data, with 40 weeks of age representing severe phenotypic difference and unneeded animal suffering. SCA7^140Q/5Q^ and WT males and females were used for all of the experiments performed. WT males and females used as controls for comparison are littermates of characterized SCA7^140Q/5Q^ mice.

### Analysis of *p16*^*Ink4a*^ and *p21*^*Cip1*^ expression

Tissue was harvested from euthanized animals, snap-frozen in liquid nitrogen, and stored at -80°C until use. Tissue RNA was extracted using the Qiagen RNeasy Mini kit, quantified on a Thermo Scientific NanoDrop, and stored at -80˚C. RNA with an A260/A280 between 1.8 and 2.2 with a concentration higher than 100 ng/μl was used for RT-qPCR. For the analysis of kidney and bulk cerebellum, RNA of sufficient quality was reverse-transcribed into cDNA using the Invitrogen SuperScript™ First-Strand Synthesis System for RT-PCR. For the analysis of heart tissue, RNA was reverse-transcribed into cDNA using the Invitrogen SuperScript^TM^ VILO cDNA synthesis kit. For kidney and bulk cerebellum qPCR, 20 μl reactions were prepared with 400 nM of each primer and 100 ng (*p16*^*Ink4a*^) or 50 ng (all others) of cDNA template. Each plate included template-negative controls without cDNA. RT-qPCR data was analyzed using the ΔΔCt method and averaged between the three technical replicates. *Gapdh* was used as an internal reference gene for all experiments. Primers used: p21_F (GCAGATCCACAGCGATATCCA), p21_R (AACAGGTCGGACATCACCAG), p16_F (CCCAACGCCCCGAACT), p16_R (GCAGAAGAGCTGCTACGTGAA), Gapdh_F (AAGGTCATCCCAGAGCTGAA), Gapdh_R (CTGCTTCACCACCTTCTTGA). Each qPCR reaction was run in triplicate. For heart tissue qPCR, Thermo Fischer Scientific Taqman probes were used: Cdkn1alpha (Mm04205640_g1), Cdkn2alpha (Mm00494449_m1), and Gapdh (Mm99999915_g1).

### SA-ßgal staining

Staining for senescence-associated ß-galactosidase (SA-βgal) was conducted following a previously described protocol [25]. Briefly, tissue samples were fixed in 10% neutral buffered formalin for 4 hours, placed in sucrose for 24 hours, and embedded in O.C.T prior to cryosectioning at 6 microns. Tissue sections were then stained with a solution containing X-gal. The pH of this solution was adjusted with NaOH and HCl as necessary to reach pH 5.9 and 6.1. Stained samples were then imaged using bright field microscopy on the EVOS XL Core Imaging System at 4× and 20× magnification.

### Tissue pathology analysis

Fresh tissue was harvested from euthanized mice and fixed in 10% neutral buffered formalin on ice for at least 24 hours. Tissue was then transferred to the Comparative Pathology Shared Resource (CPSR) at the University of Minnesota. CPSR performed paraffin-embedding, sectioning, mounting onto slides, and hematoxylin/eosin (H&E) staining using standard histological techniques. Interpretation of histological findings was performed by a veterinary pathologist.

### Urine analysis

Fresh urine was collected and snap frozen until use. 5μL of urine was used to perform a Bradford Assay to quantify proteinuria using the Protein Assay Reagent (Bio-Rad) according to the manufacturer’s specifications. Absorbance at 595 nm was measured on a Varioskan Lux (Thermo-Fisher) spectrophotometer. Bovine serum albumin (Fisher Scientific) was used to generate the standard curve.

## Results

### SCA7^140Q/5Q^ mice display renal abnormalities

Infantile SCA7 can cause nephrotic syndrome, hypertrophic cardiomyopathy, and patent ductus arteriosus, in addition to the cerebellar atrophy and retinal dystrophy characteristic of adult SCA7 [1]. However, these infantile-specific non-neural defects have not been investigated in murine SCA7 models.

**Fig 1:**
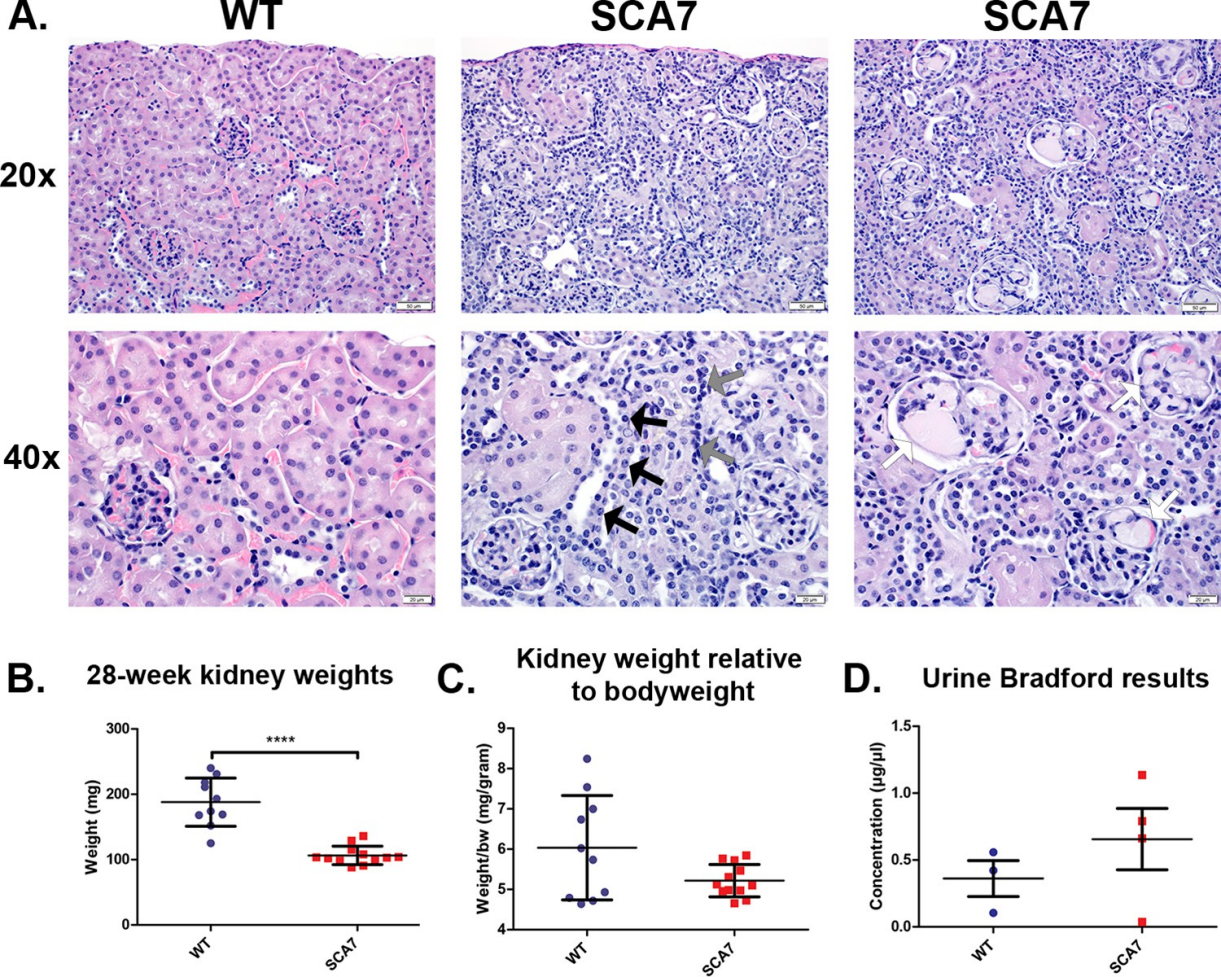
Kidney histologic and functional abnormality in SCA7^140Q/5Q^ mice. A) Kidney hematoxylin and eosin staining (H&E) of 40-week-old SCA7^140Q/5Q^ and WT littermates, B-C) absolute and relative kidney weights, and D) urine Bradford assay results of 28-week-old SCA7^*140Q/5Q*^ and WT littermates. SCA7^*140Q/5Q*^ mouse kidney demonstrates abnormal histological structure and decreased weight, likely related to kidney dysfunction. Renal hyperplasia (black and gray arrows) and glomeruli proteinosis (white arrows). Significance determined via two-tailed student’s t-test. **** *p*≤0.0001.

SCA7^140Q/5Q^ kidneys were analyzed through weight, histological analysis, and proteinuria. By raw weight, the SCA7^140Q/5Q^ kidneys weighed significantly less than WT mice (Fig 1B–1C). The mean SCA7^*140Q/5Q*^ kidney weight relative to body weight was 14% smaller than the mean WT kidney weight but did not hit the threshold for significance (6.0±0.5, 5.2±0.1, *p* = 0.051). Histological analysis showed several distinct findings present in the SCA7^140Q/5Q^ mice that were absent in their WT littermates (Fig 1A). There was evidence of tubular epithelial hyperplasia with an increased cytoplasmic basophilia and cells with an increased nuclear-to-cytoplasmic ratio. Secondarily, kidney tissue of SCA7^*140Q/5Q*^ mice showed modest numbers of immature glomeruli, 2–4 immature glomeruli were identified in the evaluation of 10 non-overlapping 400× fields from each of the SCA7 mice. In contrast, no immature glomeruli were identified in the WT mice. Additionally, mild-to-moderate multifocal mononuclear inflammation was observed within the medullary interstitium and renal pelvis. Functionally, we did not find a significant difference in proteinuria, which would indicate kidney dysfunction, in SCA7^140Q/5Q^ mice (Fig 1D). However, this negative finding could be due to limited sample size. Histopathological analysis revealed no evidence of patent ductus arteriosus or hypertrophic cardiomyopathy in this SCA7 model. Additionally, there were no obvious morphological differences in the cerebellums between SCA7 and WT mice aged 10 or 40 weeks.

### Accumulation of senescent cells in tissues affected by SCA7

A chronic buildup of senescent cells is caused by increased and unrepaired DNA damage and contributes to age-related disease. As SCA7 leads to an increased sensitivity to DNA damage, we quantified expression of cellular senescence markers *p16*^*Ink4a*^ and *p21*^*Cip1*^ by RT-qPCR.

**Fig 2:**
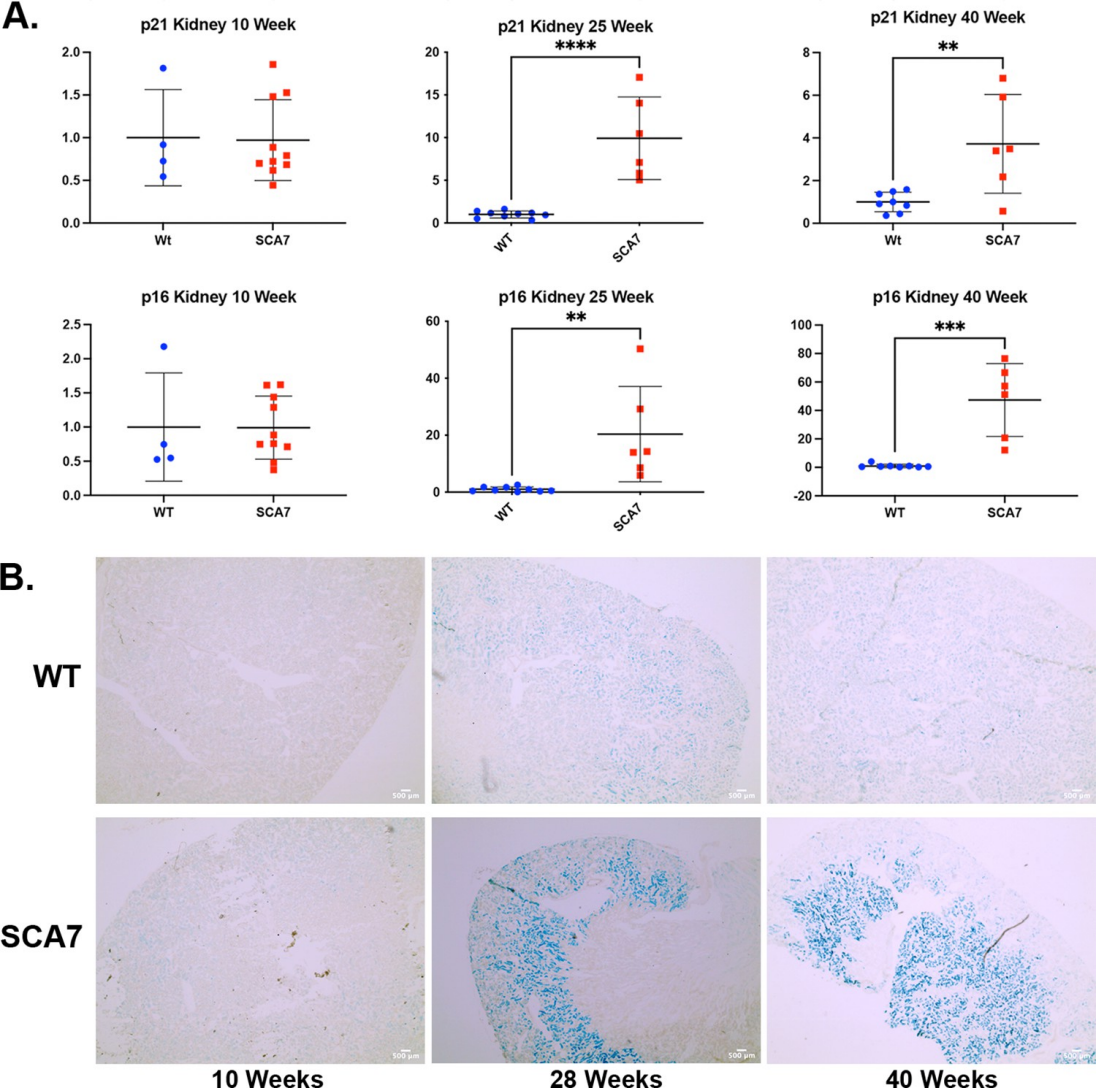
Increased cellular senescent burden in SCA7^140Q/5Q^ kidneys when compared to WT littermates. A) RT-qPCR of *p16*^*Ink4a*^/*p21*^*Cip1*^ and B) SA-ßgal staining of the kidney comparing 10-, 28-, and 40-week SCA7^*140Q/5Q*^ and WT littermates. SCA7^*140Q/5Q*^ kidney shows a markedly increased senescent profile compared to WT littermates demonstrated by RT-qPCR and SA-ßgal staining. Significance determined via two-tailed student’s t-test. ** *p* ≤0.01 via two tailed student’s t-test. *** *p*≤0.001.

At 10 weeks of age, prior to the onset of the visible ataxia scoring symptoms, there was no significant difference in the expression of *p16*^*Ink4a*^ and *p21*^*Cip1*^ in the kidneys of SCA7^140Q/5Q^ mice compared to WT controls. When p16^Ink4a^ and p21^Cip1^ were analyzed at 25 and 40 weeks, at which time the SCA7^140Q/5Q^ mice displayed a significant ataxic phenotype, both *p16*^*Ink4a*^ and *p21*^*Cip1*^ expression were significantly elevated (Fig 2A). To confirm these molecular findings of increased senescent cell burden, SA-ßgal staining was performed (Fig 2B). Increased SA-ßgal staining was present in SCA7^140Q/5Q^ mice by 28 weeks of age and severely increased by 40 weeks of age. Younger SCA7^140Q/5Q^ mice (10 weeks old) showed no increase in SA-ßgal staining (Fig 2B).

**Fig 3:**
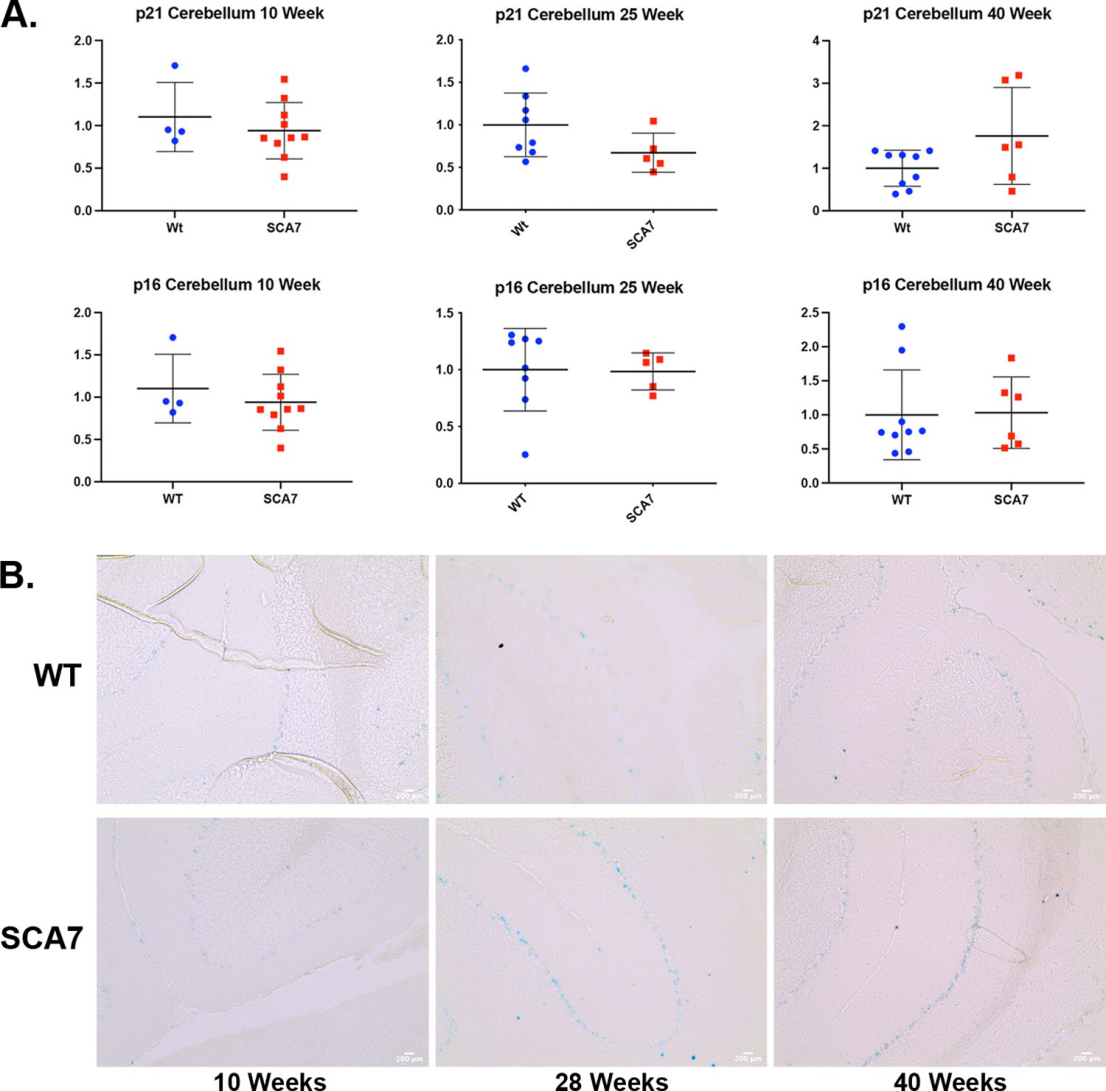
SCA7^140Q/5Q^ cerebellum demonstrates SA-ßgal positive staining without noticeable change in *p16*^*Ink4a*^/*p21*^*Cip1*^ levels. A) RT-qPCR of *p16*^*Ink4a*^/*p21*^*Cip1*^ and B) SA-ßgal staining of the cerebellum comparing 10-, 28-, and 40-week SCA7^*140Q/5Q*^ and WT littermates. Cellular senescence is demonstrated in the cerebellum of SCA7^*140Q/5Q*^ and WT mice. Statistical analysis via two-tailed student’s t-test.

We identified a potential increase in SA-ßgal staining in the SCA7 Purkinje layer of the cerebellum at 28 and 40 weeks, again after symptom onset (Fig 3B). Light SA-ßgal staining was noted in the WT Purkinje layer as well at 10, 28, and 40 weeks. We were not able to identify which cells were expressing this SA-ßgal using light microscopy. Additionally, there was no significant difference in *p16*^*Ink4a*^ and *p21*^*Cip1*^ expression in RNA isolated from bulk cerebellum of 10-, 25-, or 40-week-old SCA7^140Q/5Q^ mice (Fig 3A). Finally, the choroid plexus, which produces cerebrospinal fluid, was positive for SA-ßgal in both the WT and SCA7 mice without noticeable difference.

**Fig 4:**
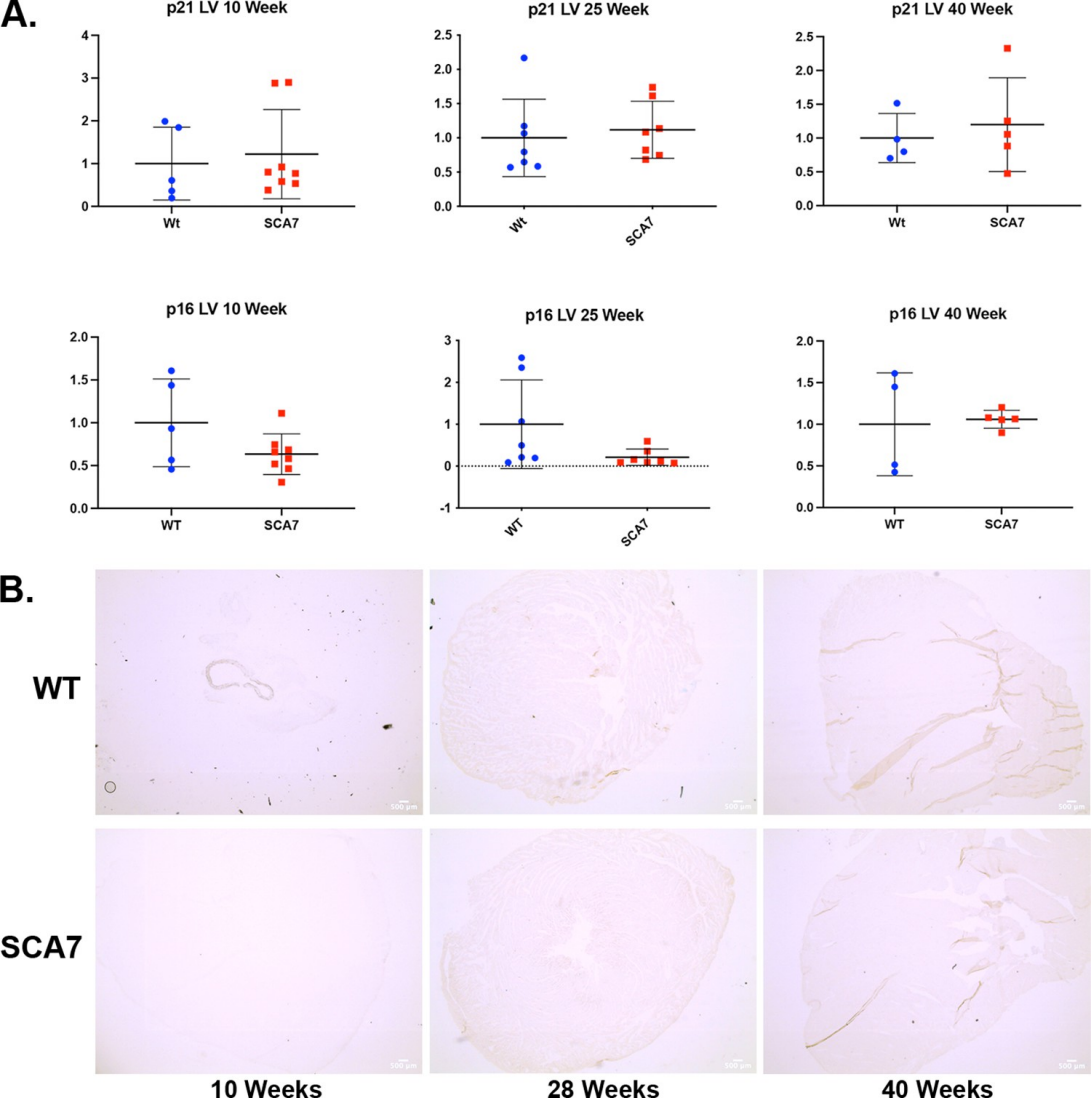
SCA7^140Q/5Q^ heart tissue shows no evidence of increased cellular senescent burden. A) Bulk RT-qPCR of *p16*^*Ink4a*^/*p21*^*Cip1*^ and B) SA-ßgal staining of the heart tissue comparing 10-, 28-, and 40-week SCA7^*140Q/5Q*^ and WT littermates. Absence of morphological differences or senescence profiles in SCA7^*140Q/5Q*^ heart tissue. Statistical analysis via two-tailed student’s t-test.

Within the heart, we found no significant differences in senescence marker expression in young or aged SCA7^140Q/5Q^ mice compared to WT controls. SA-ßgal staining in 10-, 28-, and 40-week-old SCA7^140Q/5Q^ and WT myocardium showed no significant difference in ß-galactosidase activity (Fig 4A and 4B).

## Discussion

In this study we tested the hypothesis that SCA7 results in an accumulation of senescent cells in affected tissues over the course of disease. This investigation revealed two interesting findings. First, we identified renal abnormalities that may be like those found in human infantile SCA7. Additionally, we found increased expression of senescence markers (*p16*^*Ink4a*^ and *p21*^*Cip1*^) and SA-ßgal activity in the kidney and Purkinje layer of the cerebellum of SCA7^*140Q/5Q*^ mice, indicating an accumulation of senescent cells within these tissues.

Reports of human infantile SCA7 have shown a range of glomerular and/or tubular renal dysfunction, with nephrotic syndrome being most common [1, 26]. While we were unable to definitively confirm proteinuria indicative of nephrotic syndrome in SCA7^*140Q/5Q*^ mice, the histologic pathology combined with decreased raw kidney weight in SCA7^*140Q/5Q*^ mice compared to WT indicates that SCA7 negatively affects the murine kidney. We believe the findings described here, although non-diagnostic, are indicative of renal dysfunction similar to that of human SCA7 patients.

Our data shows a correlation between abnormal renal histology and increased cellular senescence in SCA7^*140Q/5Q*^ mouse kidneys. Expression of *p16*^*Ink4a*^ and *p21*^*Cip1*^ and SA-ßgal staining confirmed increased senescent cell burden in the kidneys of SCA7^*140Q/5Q*^ mice by 25 weeks, after symptom onset, and through 40 weeks, a point at which the stage of disease is typically advanced. There was no difference at 10 weeks, prior to symptom onset. These data are internally consistent and indicate that the kidneys of these mice accumulate an increased senescent burden over the course of disease. Although this correlation between presence of cellular senescence and disease state in SCA7 is novel, it does not demonstrate causation. While further investigation is needed to determine what role senescence plays in SCA7 disease progression, previous reports have indicated that an increase in cellular senescence in various renal cell types is present in a multitude of renal diseases including IgA nephropathy, AKI-to-CKD transition, diabetic nephropathy, and autosomal dominant polycystic kidney disease [27, 28].

The histological findings of SCA7^140Q/5Q^ heart tissue did not display evidence of patent ductus arteriosus or hypertrophic cardiomyopathy found in infantile human SCA7. We did not find any difference in senescent cell accumulation between the hearts of WT and SCA7^*140Q/5Q*^ mice, in agreement with the apparent lack of pathological findings. These cardiac findings closely support the results of Yousefzadeh et al. where they examined whether expression markers of senescence with aging were tissue-specific [29]. Looking at the heart tissue of aged WT and young adult Ercc1−/Δ mice, they did not identify an increased expression of p16 ^*Ink4a*^ or p21^*Cip1*^. In fact, heart and skeletal muscle were the only tissues, out of 13 analyzed, that did not exhibit significantly increased expression of *p16*^*Ink4a*^ and *p21*^*Cip1*^. Similarly, in another study looking at *p16*^*Ink4a*^ and *p21*^*Cip1*^ expression in a *BubR1*^*H/H*^ mouse model, significantly increased expression was found in the eye, inguinal adipose tissue, and skeletal muscle, but it was not found in the heart or liver tissue [30]. The SCA7^*140Q/5Q*^ mouse model we analyzed did not present with any abnormal functional cardiac findings, unlike infantile human SCA7, and had no signs of senescence via SA-βgal staining as expected based on previous literature.

There were no obvious morphological differences in the cerebellums of these animals. These cerebellar findings are not dissimilar to previous reports. Previous characterization of the SCA7^140Q/5Q^ model did not find significant atrophy in the cerebellum. However, the Purkinje cell soma area was significantly smaller in some cerebellar lobules. Furthermore, Purkinje cells in all lobules displayed alterations in pacemaker firing rate and synapse integrity by 38 weeks [21]. It is likely that our histological analysis was not specific enough to identify these differences. Nevertheless, obvious cerebellar atrophy is commonly, but not always, reported in infantile human SCA7. Absence of obvious cerebellar atrophy may be a limitation of the models.

The most-affected location in ataxias, the Purkinje layer of the cerebellum, displayed SA-ßgal staining in the 28- and 40-week cerebellums of our SCA7^*140Q/5Q*^ mouse model. Unlike in the kidneys, we did not detect increased *p16*^*Ink4a*^ and *p21*^*Cip1*^expression that coincides with this SA-ßgal staining. However, as our RT-qPCR was of bulk cerebellum lysates, and it appears that the increased SA-ßgal activity is highly localized to the Purkinje layer of the cerebellum, it is possible that an increased senescent-like phenotype in this subpopulation of cells would be missed by RT-qPCR of bulk tissue. 28- and 40-week WT cerebellums also displayed some slight staining in the Purkinje layer. This is not unexpected, as previous studies have shown SA-ßgal staining of the Purkinje layer in 36-week WT mice, as well as positive staining in the choroid plexus [31].

Cells of the Purkinje layer may be particularly sensitive to exhibiting a senescence-like phenotype. Senescence was recently investigated in ataxia-telangiectasia, an ataxia caused by a mutation to ataxia-telangiectasia mutated (ATM), a kinase known to be a master regulator of the double-strand DNA break repair process [32]. This study found that the Purkinje layer of ATM mice displayed an increased expression of SA-ßgal that coincided with disease-specific loss of Purkinje cell volume and number. While the functional impact of this senescent-like phenotype is unknown, senescence has been reported to cause metabolic dysfunction and increase in reactive oxygenating species production, both previously reported in SCA7 [23, 33, 34]. These findings of SA-ßgal staining in this SCA7^*140Q/5Q*^ model cerebellum need to be followed up with spatial markers of a senescent-like phenotype.

In conclusion, we identified that markers of senescence increase in the kidney and in the Purkinje layer of a SCA7^*140Q/5Q*^ mouse model. These findings correlate with disease progression, but further experiments are needed to determine the cause of specific cellular senescence and the role that senescence plays in SCA7 disease progression. This could be done by selectively removing senescent cells from this SCA7^*140Q/5Q*^ mouse model with senolytics, drugs that specifically kill senescent cells. While this is the first time that senescence has been investigated in the polyQ diseases, there is some evidence that senolytics may be effective. A previous investigation looked at the impact of fisetin, now known to be a senolytic, in Huntington’s disease, another polyQ disorder [35, 36]. These results were positive, with fisetin providing functional improvement in a Huntington’s disease cell line, drosophila model, and mouse model. While the data in our study is observational and does not demonstrate causality, it opens a door connecting the polyQ diseases to our increasing knowledge of senescence. If this increased senescence is shown to contribute to disease pathology, this could lead to senescence-targeting treatments for SCA7 patients who currently have no therapeutic options.

## Supporting information

S1 Fig Significant differences of ataxia scores and weights (g) between WT and SCA7^*140Q/5Q*^ mice from 15–40 weeks of age. Values above indicate the average ataxia scores and weights ± standard deviation, observed in 5-week intervals starting from 5 weeks until 40 weeks. Significance determined using two-tailed student’s t-test. Female mice excluded from weight measurements due to not enough nonpregnant mice remaining for analysis. **p≤0.01, ***p≤0.001.
(TIF)
