# Supplementary figures and images for: Accumulation of senescence observed in spinocerebellar ataxia type 7 mouse model
Source: PLoS One. 2022 Oct 17;17(10):e0275580. doi: 10.1371/journal.pone.0275580 (PMC9576077; doi:10.1371/journal.pone.0275580)

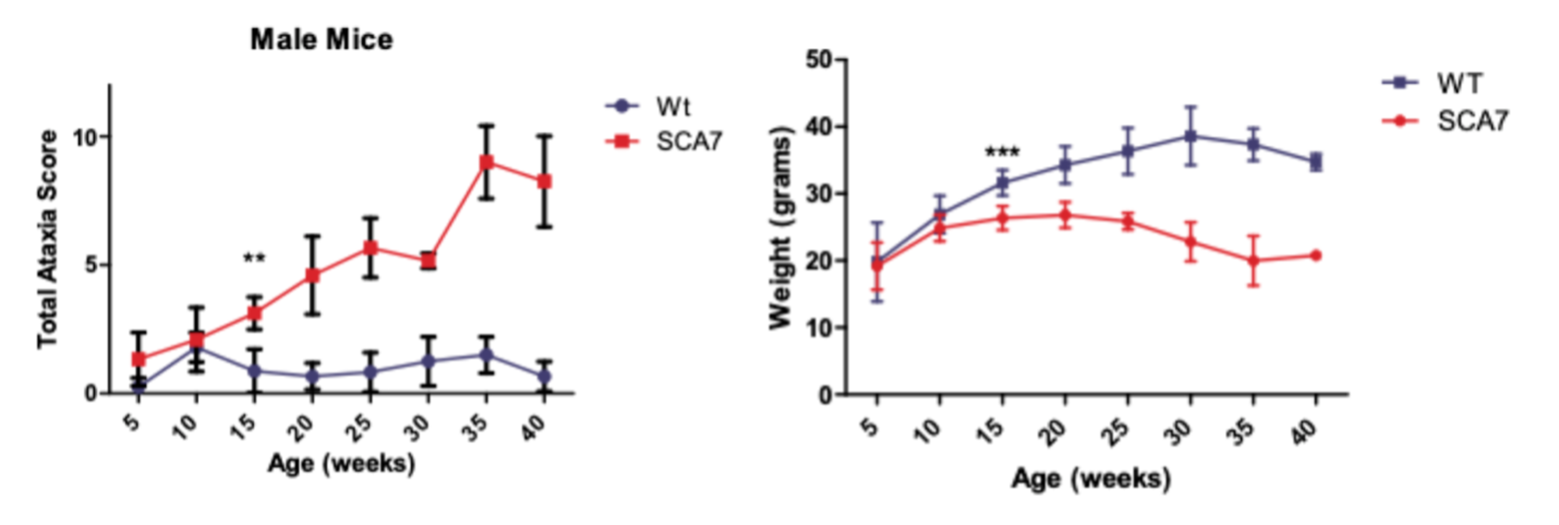

Supplement: S1 Fig — Values above indicate the average ataxia scores and weights ± standard deviation, observed in 5-week intervals starting from 5 weeks until 40 weeks. Significance determined using two-tailed student’s t-test. Female mice excluded from weight measurements due to not enough nonpregnant mice remaining for analysis. **p≤0.01, ***p≤0.001. (TIF) [file pone.0275580.s001.tif]
